# Psychedelic-assisted therapy as a complex intervention: implications for clinical trial design

**DOI:** 10.1177/20451253251381074

**Published:** 2025-10-02

**Authors:** S. D. Muthumaraswamy, M. J. Baggott, E. E. Schenberg, D. Repantis, M. Wolff, A. Forsyth, T. Noorani

**Affiliations:** The University of Auckland, 85 Park Road, Auckland 1023, New Zealand; Tactogen Inc, Palo Alto, CA USA; Instituto Phaneros, São Paulo, Brazil; Language, Action and Brain Laboratory, University College London, London, UK; Affiliated Researcher of the Project on Psychedelics Law and Regulation (POPLAR) at the Petrie-Flom Center for Health Law Policy, Biotechnology, and Bioethics at Harvard Law School, Cambridge, MA, USA; Faculty of Medicine, University of Lisbon, Lisbon, Portugal; Charité – Universitätsmedizin Berlin, corporate member of Freie Universität Berlin and Humboldt-Universität zu Berlin, Department of Psychiatry and Neurosciences, Campus Charité Mitte, Berlin, Germany; German Center for Mental Health (DZPG), partner site Berlin-Potsdam, Germany; Charité – Universitätsmedizin Berlin, corporate member of Freie Universität Berlin and Humboldt-Universität zu Berlin, Department of Psychiatry and Neurosciences, Campus Charité Mitte, Berlin, Germany; MIND Foundation, Berlin, Germany; The University of Auckland, Auckland, New Zealand; The University of Auckland, Auckland, New Zealand

**Keywords:** clinical trial design, clinical trials, complex interventions, pragmatic trials, psychedelic-assisted therapy

## Abstract

Psychedelic-assisted therapy (PAT) has typically been evaluated using conventional randomised controlled trials (RCTs), which assess treatment efficacy under highly controlled conditions. However, PAT constitutes a complex intervention, integrating pharmacological, psychotherapeutic and contextual elements that interact dynamically with patient experiences and healthcare settings. Conventional RCTs, designed for simple interventions, may fail to capture these complexities. Pragmatic trials, by contrast, evaluate interventions under real-world conditions, assessing their effectiveness across diverse clinical environments and patient populations. This position paper advocates for the application of the UK Medical Research Council’s (MRC) framework for complex interventions to the development and evaluation of PAT. This framework emphasises the necessity of articulating the underlying theory of therapeutic change, structuring intervention development into defined phases, accounting for contextual interactions and incorporating stakeholder perspectives throughout the research process. We argue that employing pragmatic trial designs, guided by the PRECIS-2 tool, will better align PAT research with the practicalities of healthcare delivery and facilitate the translation of research findings into clinical practice. Further, we address the philosophical divergence in the field between conceptualising PAT as primarily pharmacological versus psychotherapy-augmented, noting the implications of these positions for trial design and interpretation. We propose the integration of qualitative methodologies, adaptive trial designs and comparative effectiveness research to refine PAT interventions and address limitations inherent in conventional double-blind RCT approaches. Finally, we advocate for a pluralistic evidentiary model, combining academic and community-led research, to support the rigorous, equitable and sustainable development of psychedelic-assisted therapies and to avoid the historical setbacks that previously hindered progress in this field.

## Introduction

The field of psychedelic medicine and psychedelic-assisted therapy (PAT) has seen rapid expansion in the last decade. With only a small number of clinical trials conducted before 2017, one analysis of clinicaltrials.gov showed that as of May 2024, 278 clinical trials had been registered for classic psychedelics, MDMA and related compounds.^1^ Another analysis showed that as of April 2024, over 134 clinical trials for psilocybin alone had been registered studying 54 potential indications.^2^ Despite this volume of clinical trials, some of the core theoretical principles for the development of PAT as an intervention (such as the role of any accompanying psychotherapy/psychological support) remain both understudied and unresolved. In this position piece, we argue that this lack of theoretical understanding of PAT derives in part from treating PAT as a simple pharmaceutical intervention rather than as a complex intervention. We introduce and apply the UK Medical Research Council’s (MRC) well-established complex intervention framework^3^ to PAT and argue that this framework provides a more structured approach to answer not only key theoretical questions regarding PAT, but also to develop PAT in a way that will better align with existing healthcare systems and ultimately improve its utility to patients. Following this, we briefly recap some of the shortcomings of ‘standard’ explanatory clinical trials when applied to PAT and argue that PAT trials conducted in the context of the complex intervention framework could take more pragmatic design approaches. We then introduce the widely used PRECIS-2 tool^4^ for designing pragmatic trials and show how it can be applied to PAT trials, both past and present. In considering the positionality of various psychedelic research actors, we argue that academic/community-based investigators are well positioned to conduct the types of trials we envision. In particular, unlike typical drug development, the generic nature of key psychedelic drug compounds allows these types of trials to be conducted prior marketing approval being obtained from regulators. Indeed, data from the more diverse types of trials we propose could form part of a more pluralistic evidence assessment for the utility of PAT as a healthcare intervention.

## PAT as a complex intervention

Interventions are the basis of healthcare and can be defined as ‘a treatment procedure or action taken to prevent disease or improve health in other ways’.^5^ Simple interventions tend to have direct linear pathways between an intervention and outcome, while complex interventions tend to have multiple interacting components and non-linear causal pathways.^6^ The definitions of simplicity/complexity are important as the tools used to research and evaluate complex interventions can be quite different than for simple interventions where randomised controlled trials are often the ‘gold-standard’. The UK Medical Research Council’s (MRC) latest guidance for developing and evaluating complex interventions^3^ considers properties that might define an intervention as complex including ‘*the number of components involved; the range of behaviours targeted; expertise and skills required by those delivering and receiving the intervention the number of groups, settings, or levels targeted; or the permitted level of flexibility of the intervention or its components (p.2)*’. PAT typically involves preparation, dosing and integration sessions with one or more patients and therapists at a time. Delivering the intervention requires specialised clinical skills, specific therapeutic settings and flexibility in protocols – including the number and timing of sessions and the nature of therapist–patient interactions. PAT also interacts in complex ways with existing healthcare systems and is embedded within broader cultural histories of psychedelic drug use. Taken together, these features suggest that PAT fits well within the definition of a complex intervention (see also Noorani and Liebert).^7^

**Figure 1.**
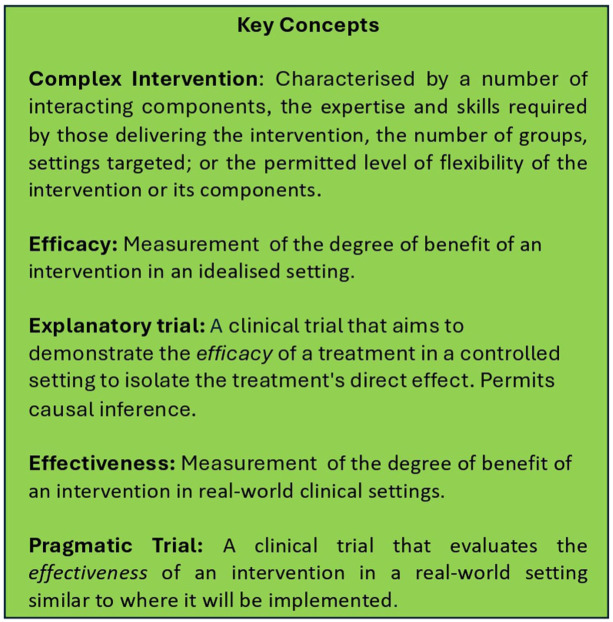
Key definitions and concepts used in the current paper. See text for further discussion.

**Figure 2.**
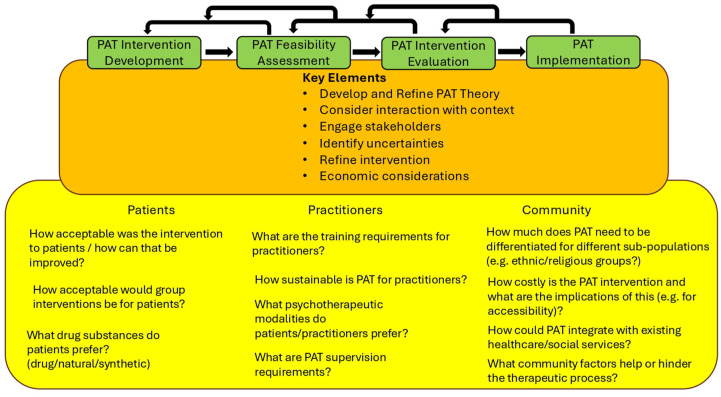
Framework for the development of complex interventions^8^ outlining the development steps and key topics to be considered at each step as applied to PAT. In the lower panel, we suggest some potential research questions. PAT, Psychedelic-assisted therapy.

The framework devised by the MRC^3,8^ challenges the view that measuring effectiveness is the primary goal of complex intervention development (See Figure 1 for definitions of effectiveness and efficacy and other key concepts discussed in the paper). Rather, the focus is broadened to topics such as improving theory and understanding how interventions contribute to change and interact with their context, which are considered equally important areas of inquiry as determining effectiveness. Complex intervention research consists of the following phases: intervention development, assessment of feasibility and design, evaluation of the intervention and its impact and implementation. These phases do not necessarily occur sequentially and progress through them may reverse as new knowledge is obtained. Six key questions can be asked at each phase: (1) How does the intervention interact with its context? (2) What is the underlying theory? (3) How can various stakeholder perspectives be included in the research? (4) What are the key uncertainties? (5) How can the intervention be refined? and (6) What are the resource consequences of the intervention? As these questions are answered, the phases of research can be progressed both forward and backward as required. Arguably, research on PAT has focused heavily on measuring its efficacy, often to the detriment of developing PAT as an intervention itself and exploring the myriad questions about how PAT might operate when embedded within existing healthcare systems and society at large. Figure 2 provides an overview of the phases of the complex intervention approach and the types of research questions that could be embedded within PAT clinical trials. We deliberately exclude effectiveness/efficacy assessment as this is the current default, and note that to answer some of these questions, outcome measures may involve surveys, questionnaires, interviews and focus groups (both quantitative and qualitative data). Many of the research questions displayed in Figure 2 could be answered by prospective cohort (open-label) studies. That said, in the following sections, we outline how it would be desirable for PAT researchers to embed some of these questions within pragmatic randomised controlled trials. We next provide further motivation for conducting such trials, given the limitations of interpreting data from psychedelic double-blinded randomised controlled trials (DB-RCTs).

## The limitations of randomised controlled trials

DB-RCTs provide the best evidence for determining whether a non-complex treatment has a causal effect on outcomes. This is why they are considered the gold standard for many areas of research. We have previously written extensively on the theoretical basis for the randomised control trial based on the Rubin causal model and its underlying assumptions of positivity, consistency, exchangeability and non-interference, and do not recapitulate those details here^9,10^). Even if all the assumptions of the RCT are met and the study has been perfectly controlled and executed, the knowledge gained from RCTs is easily misinterpreted/over-extrapolated as discussed by Deaton and Cartwright.^11^ First, while randomisation is often thought to create balance between treatment and control groups, it actually only ensures balance in expectation over multiple trials, meaning individual trials may still suffer from imbalances, particularly in trials with smaller sample sizes or with numerous covariates. Second, although RCTs aim for unbiased estimates of the average treatment effect (ATE), unbiasedness does not guarantee precision; results can be highly variable between trials, leading to imprecise conclusions. Third, even when RCTs produce unbiased estimates of the ATE, this information alone may have limited practical value, especially when treatment effects vary widely across individuals or settings. The practical value of the ATE to predicting individual effects is limited because it represents the central tendency of a distribution of individual effects and it is not possible to know where any individual falls within that distribution. Similarly, even if the ATE = 0 in a sample, this does not mean that the treatment works for no one – there may simply be an unidentified sub-population that the treatment does work for – but the parameters that define this sub-population cannot be identified. Both these interpretative mistakes are created by misunderstanding the fundamental problem of causal inference (see refs. 9, 10, 12 for more detailed discussion). Furthermore, as pointed out by Kaptchuk,^13^ double-blinding in RCTs is not a neutral device as it can change the mindset of patients, potentially causing biases such as ambivalence, passivity, confusion, resentful demoralisation and voluntary submission. In fact, the ‘uncertainty’ created in patients by being blinded to the intervention may well modify treatment effects/therapeutic processes and this has received little explicit study in the psychiatric literature.^14^ Other biases present in RCTs include those caused by the informed consent process, investigator self-selection, Hawthorne effects and outcome measurements affecting the intervention.^13^ Taken together then, there are many reasons why the ATE measured in an RCT is a far from perfect representation of any underlying treatment effect.

Importantly, in the context of the current work, RCTs often struggle with external validity – that is, the validity of applying a study’s conclusions to other settings, healthcare contexts, patient populations and so forth. The extrapolation fallacy of assuming that the effects obtained by RCTs simply extrapolate to other contexts,^11,15^ may be a major issue for complex interventions including PAT. By contrast, internal validity refers to the validity of conclusions drawn within the context of a particular study. Typical explanatory RCTs with their tightly defined treatment protocols, eligibility criteria and other somewhat contrived features are said to prioritise internal validity at the expense of external validity (*cf* later with pragmatic trials).^15^ However, the internal validity of PAT trials is highly questionable given the notably high frequency of functional unblinding that occurs.^12,16,17^ These functional unblinding effects likely cause over-estimates of the ATE found by PAT trials. This creates the uncomfortable situation in which existing PAT trials may lack both internal and external validity. The remedy for this situation may be to think more expansively about what PAT research could involve in terms of complex intervention design as previously outlined, while also incorporating principles of pragmatic RCT design.

## Study design and inferential statistical considerations

**Figure 3.**
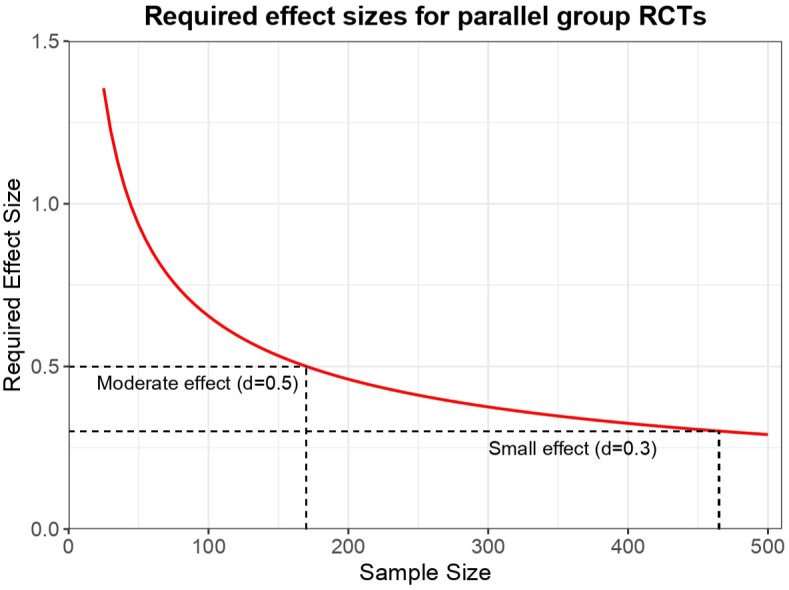
Required effect sizes for parallel-groups RCTs as a function of overall sample size. A sample size of 170 is required to achieve sensitivity to a moderate effect size (*d* = 0.5), while a sample size of 465 is required to achieve sensitivity to small effect sizes (*d* = 0.3). RCT, randomised controlled trials.

PAT is a complex intervention and, as currently formulated, is heavily resource-dependent. Obtaining even a single data point in a PAT study is resource-intensive and trials have rarely exceeded 100 participants. For reference, the two phase III MDMA-AT studies conducted to date included 90^18^ and 104 patients^19^ – relatively small for phase III studies. As currently instantiated, we argue that PAT studies do not lend themselves easily to quantitative evaluation based on purely inferential statistical principles. Consider the generic power calculations for a study aiming to evaluate the differences between two groups (e.g. a parallel groups RCT). Entering standard parameters (with α = 05, (1–β) = 0.9, two-tailed) the required sample size as a function of effect size is plotted in Figure 3. It can be seen that for a small effect size of *d* = 0.3 a sample size of 465 is required and for *d* = 0.5 a sample size of 170 is required. Notably, standard antidepressant effect sizes have been found to be ~0.3, while an effect size of 0.5 is a moderate size. While some PAT trials have shown large effect sizes, these are likely over-estimates due to functional unblinding^12^ and given the preceding logic, most PAT trials are probably only realistically powered to detect ATE estimates biased by functional unblinding. As such, almost all PAT trials, especially investigator-initiated trials, are likely to be underpowered in terms of having a singular focus on determining statistical significance for efficacy outcomes.

Should we abandon PAT clinical trials then? No. Increased emphasis on non-efficacy/effectiveness outcomes, descriptive statistics and qualitative outcomes would allow more numerous, smaller trials to more rapidly determine what works. Notably, qualitative research can often achieve data saturation (sufficient data to draw conclusions) at much smaller sample sizes (e.g. 9–17 participants^20^). Such approaches would allow treatment protocols to be adjusted during a single study (e.g. in an umbrella protocol) or across multiple studies to allow faster optimisation of PAT factors. While overall evaluation of the efficacy of PAT may be a desirable long-term goal, it may be premature given the many unanswered questions regarding how complex PAT interventions might be implemented.

One suggested design to potentially decouple drug from therapy variables that we and others have proposed is the 2 × 2 design.^12,21,22^ However, statistical considerations here are even more intimidating. Using power calculations like those presented above to detect a small interaction effect (*d* = 0.3) would require a total sample size of *n* = 922 and for a moderate effect size *n* = 338. From a pragmatic perspective, such samples are simply not realistic, and so this design should probably not be considered feasible.

## Pragmatic versus explanatory RCTs (effectiveness vs efficacy)

In a landmark paper, Schwartz and Lellouch^23^ distinguished between pragmatic and explanatory approaches to clinical trials, where the explanatory approach is aimed at understanding whether a difference exists between two treatments, often in idealised circumstances, while the pragmatic approach seeks to compare treatments under the conditions in which they will be applied in clinical practice. They give an example of testing the effect of an anti-cancer drug to be delivered for 30 days to sensitise the patient to subsequent radiotherapy. In an explanatory trial, the control group would wait (or be given a placebo) for 30 days and then be compared with the treatment group. However, in a pragmatic real-world trial, the control group would be given radiotherapy immediately, like they would in real-life practice. The explanatory trial seeks to understand whether the anti-cancer drug ‘works’ while the pragmatic approach determines how much difference it will make in real-life treatment. The pragmatic trial thus answers the criticism of clinicians that the research establishment often fails to provide answers to relevant clinical questions.^24^ Based on these approaches, policymakers and trialists distinguish between the efficacy and effectiveness of an intervention. Explanatory trials aim to estimate efficacy – whether the intervention works under idealised circumstances while pragmatic trials aim to determine effectiveness – the degree of benefit in more real-world clinical settings.^25^ That said, the definitions of efficacy versus effectiveness are not yet universally agreed upon. Most notably, the Food and Drug Administration (FDA) in their guidance documents for evaluating the evidence regarding drug products in general ^26^ and for psychedelic drug development specifically^27^ use the terms efficacy and effectiveness interchangeably (see also Schenberg^15^). Indeed, seen through the lens of pragmatic/explanatory trials, the FDA Psychopharmacologic Drugs Advisory Committee^28^ when asked to evaluate the evidence of effectiveness (not efficacy) of MDMA-AT were in fact evaluating its efficacy as the MDMA-AT phase III trials^18,19^ evaluated tested the efficacy of MDMA-AT (not its effectiveness).

**Figure 4.**
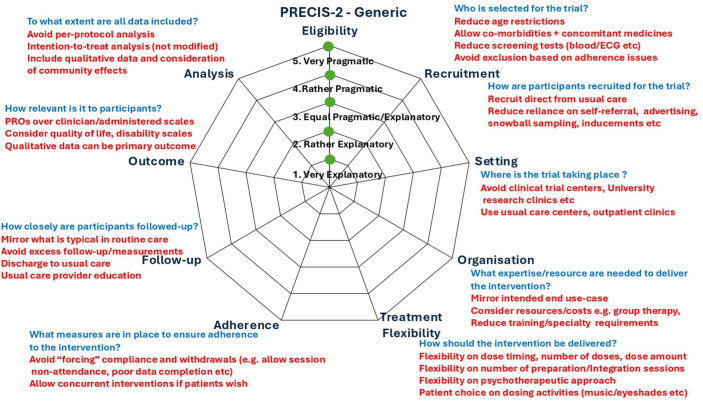
Schematic of the PRECIS-2 criteria for the assessment of how pragmatic a clinical trial is.^4^ The standard criteria are in black/blue text, while considerations for each domain that may be important for PAT trials are shown in red text. PAT, psychedelic-assisted therapy.

It is important to bear in mind that pragmatic and explanatory trials are not a dichotomy but sit on a continuum.^29^ The PRECIS-2 (PRagmatic Explanatory Continuum Indicator Summary) tool^4^ which supersedes the PRECIS tool^30^ provides a formal framework for trial designers to assess how pragmatic or explanatory their trial design is across nine domains (see Figure 4), providing a method to quantify where trials exist on the explanatory/pragmatic continuum. These domains are essential for evaluating the applicability of trial results to real-world settings. The tool focuses on ensuring that the trial design aligns with the intended use of the intervention, making trials fit for application to routine clinical practice. Each PRECIS-2 domain can be scored using a Likert scale from one (very explanatory) to five (very pragmatic). For a given trial, the sum score of the domains gives an overall indication of how pragmatic the trial is (ranging from 9 to 45). While a previous perspective piece has called for increasing use of pragmatic trials for the development of psychedelic medicine^31^ detailed consideration for how this might be instantiated was not elucidated. Below, we consider each of the PRECIS-2 domains and propose variables that might influence the evaluation of each domain with respect to PAT. We then apply these criteria to existing PAT trials to demonstrate that the focus of previous trials has been largely explanatory, reflecting the current evidentiary requirements of drug regulators for medicines to be approvable.

### Eligibility

The eligibility criterion reflects how similar the trial participants are to those who would receive the intervention in real-world settings.^4^ In the case of PAT trials to date, even for those diagnosed with the target disorder, has been highly restricted, and CONSORT diagrams for PAT trials typically show low admission rates even among those with the target disorder. Allowing a wide range of participants without exclusion based on age or potential adherence issues would better reflect clinical practise, as would increasing acceptance of co-morbid disorders that are often included in clinical settings. For example, excluding patients with substance use disorders in a depression/PTSD study is not required. Removing screening tests (e.g. bloodwork or 12 lead ECG) that may not be available/unlikely to be used in routine practice would increase the PRECIS-2 score.^4^ Finally, many existing PAT trials exclude patients based on concomitant medications or insist that patients taper off these medications prior to the participant receiving PAT. Generally, patients/services would prefer not to taper patients off medicines as this potentially requires extra management and stress for patients. In one of our studies (SM^32^), as part of a patient co-development process, 85% of 46 survey respondents felt that patients should decide whether they taper off medication before entering into the trial. Safety is of course an issue but there is increasing evidence around interactions of psychedelics with other common psychiatric medicines and classical psychedelics in particular seem to have a good potential safety profile in this regard^33^ while MDMA trials may need to be more restrictive with concerns regarding drug–drug interactions.^34,35^ There is also the potential for concomitant medications to blunt therapeutic drug effects^34^ but this might be mitigated by pragmatic personalised dosing protocols. Investigators may also wish to consider whether exclusion based on family history of psychosis is warranted.^36^ Overall, broadening inclusion/exclusion criteria to allow a more real-world participant pool that mirrors the typical clinical population will increase the external validity of obtained data.

### Recruitment

For pragmatic trials, recruitment should resemble the way patients would typically enter care in real life.^4^ For most PAT trials to date, recruitment is typically based on advertising to potential participants or where existing patients of study team members are enrolled. The former is particularly problematic as the subset of participants motivated enough to proactively seek information about enrolling in a clinical trial with high demand characteristics is quite possibly a biased selection of the overall eligible population. This population is likely to have a higher expectancy bias and may exaggerate positive outcomes relative to the total population.^9^ For pragmatic trials, reducing reliance on recruitment methods like self-referral or snowball sampling and recruiting directly from routine care settings such as clinics or hospitals would ensure that the participants are representative of those who would require treatment in everyday clinical practice.

### Setting

The setting of the trial should closely match where the intervention would be delivered in the real world. Loudon et al.^4^ note that selecting sites amongst specialist/academic centres is likely to reduce the PRECIS-2 score in this domain. For PAT, conducting the trial in usual care settings like outpatient/community clinics, rather than specialised research centres, ensures the environment is reflective of where the treatment would normally occur, making the findings more applicable to routine practice. During the intervention design process, trialists should consider what the end-use setting for PAT will be rather than conducting trials in specialist centres and assuming that the results will transfer to end-use settings. If PAT is intended to be delivered in novel and/or bespoke sites, these could be utilised in pragmatic trials, but the resulting findings would be bound to include the costs of setting up such sites in the intervention’s economic forecasting (see next point).

### Organisation

The organisation criterion focuses on the resources and expertise required to deliver the intervention. In PAT, it is critical to consider the resources required to deliver the intervention – particularly around the costs that would be associated with the treatment. For example, MDMA-AT as conducted in the Lykos phase III trials has been estimated to have a direct cost of US$11,537 per patient^37^ and similarly private clinics in Australia in their authorised psychedelic prescriber scheme are pricing PAT at ~AUD$25,000 per patient. This level of expense may be more than many are able or willing to afford. Most of the costs of the treatments are due to the training/specialist requirements dictated for the supervising clinical personnel. Modelling suggests that implementing group therapy protocols can significantly reduce the cost per patient.^38^ In a pragmatic (and complex intervention) approach to this issue, trialists should develop the PAT protocol with realistic real-world resourcing in mind and then apply these in trials such that the data will more directly reflect real-world application.

### Treatment flexibility

Treatment flexibility refers to how adaptable the delivery of the intervention is in the trial. For PAT, allowing flexibility in aspects such as dose timing, number of sessions and the level of the dose would be appropriate. There is also the question of how regimented the psychotherapeutic support should be, given that in the real world, therapists are likely to use their own clinical reasoning/judgement for different patients and deviate from strictly manualised therapy. Further, patient preferences (e.g. use of music or eye shades) during dosing as well as including options for remote access for preparation/integration sessions should be considered.

### Adherence

Adherence measures should reflect the flexibility of real-world settings, where strict adherence to rigid protocols may not be enforced. In an explanatory clinical trial, participants may be required to comply rigidly with the treatment protocol. Non-attendance or incomplete data collection might lead to withdrawal of the patient from the study using pre-defined criteria, which is unlikely to be the case in clinical care. Also in clinical trials, typically patients are strictly prohibited from seeking concurrent interventions; however, practical realities of everyday real-world patient engagement are that they may undertake concurrent interventions that do not directly impact PAT and as such these should be considered permissible.

### Follow-up

Follow-up intensity should align with standard care practices, avoiding excessive follow-ups that would not typically happen outside of a trial. For PAT, this means following patients in a manner similar to routine care, such as discharging them back to their usual care providers without extra follow-up visits or unnecessary measurements. It may be that education for usual care providers is required such that they understand the PAT intervention that their patients have undergone.

### Primary outcome

The primary outcome should be meaningful to participants and relevant to real-world care. As such, focusing on patient-reported outcomes (PROs) like quality of life and disability, rather than clinician-administered scales, ensures that the results are directly relevant to patients’ experiences, aligning with what is important to them in routine practice.^39^

### Primary analysis

The primary analysis should include all data from the trial, using an intention-to-treat approach to reflect real-world variability in preference to per protocol or modified intention-to-treat (all participants who receive the intervention). Full intention-to-treat analysis is preferred over modified intention-to-treat because in pragmatic trials dropouts and dropout rates provide important information about the real-world effectiveness of the intervention. In the early phases of PAT development/implementation, extensive use of qualitative data might be appropriate to aid with intervention development and may indeed be more useful than quantitative data. The use of qualitative data potentially allows for more rapid cycling through the intervention development process, and the use of qualitative data indicates a move beyond a simple pass/fail assessment of clinical trial data.

## How pragmatic are existing PAT trials?

**Figure 5.**
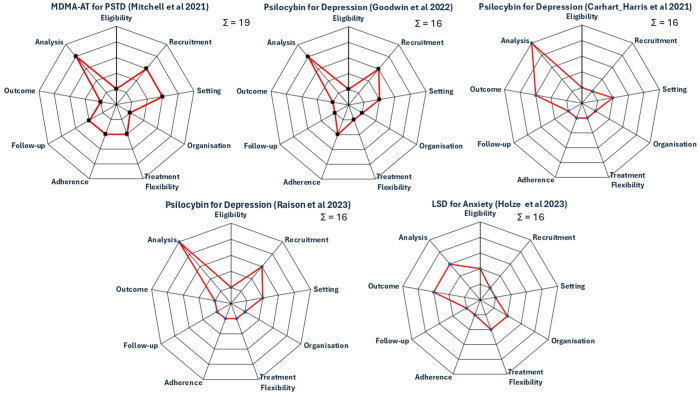
PRECIS-2 Evaluation of a set of major trials that have been conducted in PAT to date including a phase III trial of MDMA-AT for PTSD,^18^ several phase II trials of psilocybin for depression^40–42^ and a phase II trial of LSD for anxiety.^43^

In this section, three authors (SM, AF, TN) applied the PRECIS-2 criteria to a selection of existing PAT trials. This account is not intended to be exhaustive, and we focus on some of the major research trials and indications that have been published in PAT to date. In particular, we include one of the phase III Lykos trials of MDMA-AT for PTSD,^18^ phase II RCTs of psilocybin treatment for depression ^40–42^ and a phase II trial of Lysergic acid diethylamide (LSD) for anxiety disorders.^43^ The results of these are displayed in Figure 5. As can be seen, the PRECIS-2 scores for psychedelic RCTs are low, typically around 15–16, which is heavily skewed towards the explanatory side of the explanatory–pragmatic continuum. One counterargument is that, to some extent, existing PAT trials are in fact more pragmatic than how they are being presented. For example, trial publications often present schematics of the ‘ideal’ trial for participants – specifying the exact number of preparation/treatment/integration sessions. The degree to which trialists actually deviate from these schematics is not clear and almost never reported and indeed we (SM, AF) have previously called for greater transparency of reporting regarding what actually happens in PAT trials – this should include transparent reporting of the number and extent of protocol deviations.^12^ A second possible counterargument is that it is simply not possible at this stage to develop a PAT clinical trial that is pragmatic. However, in our (SM, TN, AF) experience, this is not correct. In a phase II PAT protocol that we are developing, our PRECIS-2 self-evaluation for the trial achieved a score of 37, which is heavily skewed towards the pragmatic side of the continuum. We note that in most pharmaceutical development, new drugs are under the control of pharmaceutical companies and investigators cannot begin truly independent pragmatic trials until regulatory approval for the drugs is obtained and pragmatic Phase IV trials can then begin.^44^ For PAT however, the major drugs of interest (e.g. psilocybin, MDMA, LSD) are available to Good Manufacturing Practise (GMP) standard from generic manufacturers, allowing pragmatic trials to be used earlier in the PAT development process. This is important as the purpose of pre-authorisation explanatory drug trials is to achieve product authorisation – not to provide practical measures of effectiveness, nor any understanding of potential therapeutic mechanism or underlying process of therapeutic change.^44^

Pragmatic trials clearly have limitations on their interpretation. For example, a treatment assessed pragmatically in one region does not guarantee its transferability to another location/setting,^45^ thus they are also vulnerable to the extrapolation fallacy. Moreover, it is not guaranteed that increasing study heterogeneity within a study necessarily increases the external validity of the study.^45^ Pragmatic trials also have the limitation of generally not being able to identify placebo effects from treatment effects, and when results are non-significant, there is a much broader parameter space that could potentially explain null effects (for both efficacy and safety data). Because of this, pragmatic trials alone are not able to distinguish drugs with a causal role in a therapeutic effect from ‘snake oil’. As such, it is critically important to view pragmatic and explanatory trials as complementary evidence-gathering tools. That said, the safety data from pragmatic trials is a much more realistic representation of real-world safety data and more likely to pick up adverse events, particularly given the more heterogeneous samples that are used. At the same time, from a health improvement perspective, a pragmatic trial demonstrating safety and effectiveness provides strong evidence for its own continuation, adjustment and even expansion as a treatment option that is already locally embedded.

## Complex interventions, PAT theory and implications for research design

One of the key elements of the complex intervention approach is to stress the importance of having a theory of change as to how an intervention works, what the key components of the intervention are and how these components might interact.^3^ In this regard, one could argue that PAT has neglected exploring the theoretical basis and necessary elements of PAT at the expense of efficacy testing. Most notably, a recent split emerged in the psychedelic literature between those therapeutic approaches that view the psychotherapeutic aspects of PAT as integral to the therapeutic process^46–48^ as opposed to those that view only minimal ‘psychological support’ as required to ensure patient safety^49^ (perhaps PAT vs psychedelic therapy (PT)). Gründer et al.^50^ point out that in practice this distinction is fuzzy as even in PT frameworks, the essential psychological support provided is a form of psychotherapy. Remarkably, while the largest study to date testing psilocybin for depression referred to the treatment as ‘psilocybin administered with psychological support’^41^ a preceding publication outlining the same industry-led study’s therapist training programme describes ‘psychological support’ as a complex and structured set of psychological interventions, including highly process-directive psychotherapeutic techniques provided by experienced mental health professionals throughout preparatory, dosing and integration sessions.^51^ Although the authors avoid referring to their ‘psychological support’ model as psychotherapy, they explicitly acknowledge that it integrates psychotherapeutic models such as cognitive behavioural therapy, acceptance and commitment therapy and mindfulness-based therapy. One might then argue that the attempt to distinguish PT from PAT does not primarily reflect distinct theoretical assumptions about how psychedelics work. Rather, considering that regulatory bodies such as the FDA do not regulate psychotherapy, industry players’ ongoing efforts to portray psychedelic therapy as anything but psychotherapy can be understood as a strategy to obtain regulatory approval for specific psychedelic drugs to be marketed.^52,53^

While the distinction between PAT and PT may be fuzzy and is perhaps more often made for strategic rather than theoretical, practical or clinical reasons, it can, at least in principle, emerge from genuinely distinct philosophical positions on how psychedelics might work therapeutically and the underlying theories of change. The traditional DB-RCT framework is not at all concerned with the theory of how an intervention works, and all too often, trialists do not clearly specify what the core elements of their therapeutic approach are. However, we argue the philosophical position on PAT/PT greatly impacts how trials ought to be conceptualised and designed. To the theorist who hypothesises that most of the treatment’s efficacy is attributable to the drug alone, while adjacent psychological support is a mere safety measure that does not contribute to efficacy (PT), traditional drug development designs seem most appropriate – for example, parallel groups RCTs with placebo, active placebo and dose–response control conditions. It behoves such theoretical approaches to grapple with issues such as expectancy and functional unblinding (see Aday et al.^54^ and Muthukumaraswamy et al.,^12^ for detailed consideration and trial design suggestions). However, even with psychological support models that are more clearly differentiated from psychotherapy than has been the case in studies to date – for example, by employing ‘psychological supporters’ who are not in fact trained psychotherapists – such designs cannot empirically test the fundamental theoretical assumption that psychological support (or its interaction with drug effects) is unimportant to treatment efficacy. To effectively examine this assumption, study designs more commonly used in psychotherapy research would be required. In this sense, critically examining the role of psychotherapy in psychedelic therapy, Goodwin^55^ noted that ‘it may be helpful if studies of hallucinogens are not thought of as drug studies at all, but as psychological treatment studies. (p.1201)’.

On the other hand, for the theorist who views psychedelic therapy as psychedelic-augmented psychotherapy, the central question addressed by DB-RCTs, that is, whether a drug is effective in treating a mental disorder, is inherently flawed. More aligned with the view that psychedelics are ‘catalysts’ of psychotherapy – a perspective widely upheld throughout the medical history of psychedelics – is the question of whether these drugs can induce or enhance psychotherapeutic processes. From this, a range of critical questions emerge concerning the optimisation of psychedelic therapies. For example, under what circumstances, and for whom, are psychedelics most likely to induce psychotherapeutically valuable experiences? What characteristics of an ongoing psychotherapy process indicate the optimal timing for scheduling a psychedelic session? Is it possible to purposefully influence the likelihood of specific sub-types of psychotherapeutically valuable psychedelic experiences by modulating drug-related factors (e.g. dosage, type of substance, or mode of administration) or contextual factors (e.g. preparatory interventions or different dosing settings)? What psychotherapeutic methods are most effective in harnessing therapeutic benefits from specific types of psychotherapeutically valuable psychedelic experiences? Although some hypothetical answers to such questions may be derived from psychotherapeutic theories and |conventional (non-psychedelic) psychotherapy research, such questions remain to be examined. It can therefore be surmised that RCTs to date have likely tested sub-optimal forms of PAT and reveal little information on how to optimise them.

Thus, for the theorist who believes that psychotherapy and its interaction with drug effects are critically important to the efficacy of psychedelic treatments, psychotherapy research designs are probably more appropriate than DB-RCTs. Schenberg^56^ advanced the philosophical position that attempting to blind PAT studies is futile since these overtly consciousness-altering drugs profoundly affect the perceptions, thoughts and feelings of the patient. Furthermore, the distinction between these two positions imply two different causal claims, which further influence all stages from study planning, execution and data interpretations.^15^ Indeed, if the endpoint of the psychedelic administration is to create a malleable state that the psychotherapist can work with, then the closer an active placebo becomes to mimicking this state, the more it will itself promote efficacious psychotherapy. This critique of the DB-RCT methodology is similar to that raised by original psychedelic researchers Hoffer and Osmond.^57^ This is a reasonable argument but, being axiomatic, difficult to falsify.

**Figure 6.**
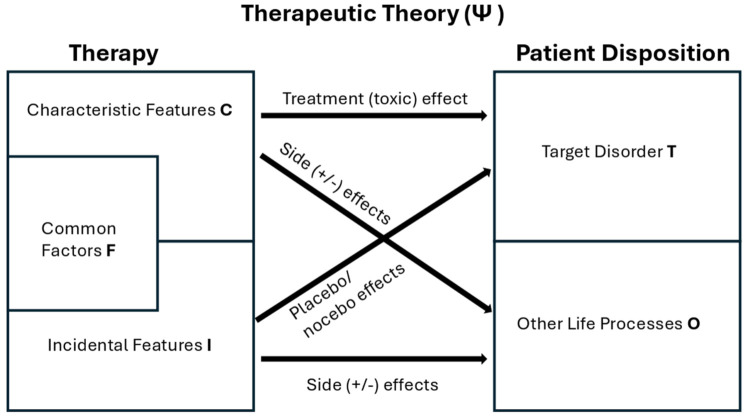
Conceptual framework for development of a therapeutic theory adapted from Howick^59^ and Grunbaum.^58^ Common factors are inserted in a neutral position at the discretion of the theorist, however, in our view, as per Blease^61^ common factors should be regarded as integral components (characteristic features) of the PAT approach taken. PAT, psychedelic-assisted therapy.

A key principle though is for investigators to centre their therapeutic theory in the clinical trial design process rather than forcing the method for evaluating it (e.g. the DB-RCT) onto how it is evaluated – see also Schenberg^15^ who arrives at a similar philosophical position but through different argumentation). The therapeutic/placebo theory of Grunbaum^58^ modified by Howick^59^ provides a useful backdrop (see Figure 6). The advance Grunbaum made was not to conceive of a treatment/placebo based on its physical characteristics, but to embed the treatment within a therapeutic theory. The therapy itself can then be divided into its *characteristic features* (causally relevant to the mechanism of action) and *incidental features* (not causally relevant to the mechanism of action, but nonetheless perhaps associated with outcome). This theoretical approach allows the placebo/treatment approach to be extended beyond the study of drugs to other interventions including psychotherapy.^60^ Within the context of PAT/PT, theorists could define these features. For example, a PT theorist might define provisioning of psychological support as an incidental feature alongside music and eyeshades, whereas a PAT theorist might define these as characteristic features. Another distinction between PAT and PT that might be made is that PT can provide explanations with a simple linear causal framework (treatment to response), whereas the more complex PAT intervention can be envisioned as a dynamical (patient/drug/therapist/environment) system.

When applied to PAT, the issue arises of how to differentiate characteristic and incidental features for a particular psychotherapeutic modality – specifically where ‘common factors’ should be placed. Common factors are those factors shared by many types of psychotherapy such as goal consensus and collaboration, therapeutic alliance, empathy, positive affirmation, congruence/genuineness and expectation amongst others.^62^ More process-oriented conceptualisations of common factors such as Grawe’s ‘general change mechanisms’^63,64^ also specify distinguishable types of therapeutic experiences that are shared by all effective psychotherapies. These factors are just as present in PAT as in other psychotherapies and presumably contribute to clinical response.^65,66^ Regarding the placement of common factors in the Grunbaum model, some authors have categorized them as incidental.^67^ Others have pointed out that ‘common factors’ should be considered characteristic features since they are commonly seen as integral treatment components in psychotherapeutic theories.^61^ Note that common factors can potentially be manipulated in a trial context and, importantly, PAT/PT theorists can define the placement of common factors according to the therapeutic theory they propose and test.

Following this argument, the design of PAT trials would then look more like trials conducted for psychotherapy than pharmaceutical trials – with the choice of control conditions being the major difference with such trials. Gold et al.^68^ provide a pragmatic and useful decision framework for selecting control conditions for randomised trials of behavioural interventions, considering factors such as participant risk, resource requirements, trial size and anticipated effect size. Briefly, for smaller phase II trials treatment-as-usual, no treatment and waitlist controls are generally more appropriate, whereas non-specific factor controls/dismantling studies and active comparator studies require larger sample sizes, typically anticipate smaller effect sizes, and are more appropriate for later phases of research.^68^ There are a number of other detailed sources specifying pros and cons for various control conditions in psychotherapy research^69–72^ and their effect on outcomes^72^ which the reader is referred to rather than repeating the details here. That said, it is worth bearing in mind that there has been long disagreement in the psychotherapy literature about the ‘dodo bird verdict’ – the hypothesis that all validated forms of psychotherapies produce similar outcomes regardless of their specific components.^62,73^ As pointed out Langlitz and Gearin^22^ even if forms of psychotherapy are therapeutically equivalent that does not mean they are ethically equivalent or indeed appropriate for particular clients when put in the context of PAT.

Based on this, some have argued that rather than ‘tilting at windmills’ in psychotherapy research evaluating the efficacy of psychotherapy there is no need to control the placebo effect.^74^ Kirsch et al. argue that in psychotherapy research, no-treatment or usual care is the most appropriate control condition since dismantling studies, additive RCTs, and meta-analyses generally fail to show significant effects of specific hypothesised therapeutic components. Given that some recent critiques of PAT have come from researchers working in psychotherapy^75–77^ it is worth reflecting on Klein’s^78^ contention ‘*The bottom line is that if the Food and Drug Administration (FDA) was responsible for the evaluation of psychotherapy, then no current psychotherapy would be approvable* (p.84)’ still holds true. Quite probably since such trials are generally unblinded,^79^ infrequently carefully document adverse events,^79–81^ and often lack rigorous intervention standardisation and fidelity reporting^82,83^ compared to pharmaceuticals (the purpose of GMP), or trial oversight standards (external Good Clinical Practice monitoring and auditing).

Related to pragmatic trials, comparative effectiveness (CE) research – trials directly comparing active treatments as per usual practise – are important for helping clinicians and policymakers to make good decisions.^84^ At the later stage of complex intervention development for PAT, large scale pragmatic CE trials would be particularly helpful and provide useful complementary evidence. To reduce blinding and allegiance biases such trials could involve cluster randomisation (by study site) or randomisation to treatment prior to informed consent being given. There is a good argument for pharmaceutical companies and regulatory authorities performing CE trials as part of their phase III research programmes. If one accepts the premise that it will be difficult to conduct phase III PT trials that are not affected by functional unblinding (or desirable if one takes the PAT approach), there may remain uncertainty as to whether treatment effects can be conclusively identified from placebo effects in these trials.^10^ Simply conducting more trials will not resolve the identification issue. For example, the second MAPS phase III for MDMA-AT trial for PTSD^19^ simply repeated the trial design of the first phase III trial^18^ and did nothing to resolve the treatment-effect identification issue.

Looking forward, publicly available information regarding the Compass Pathways^85^ and Cybin^86^ phase III psilocybin for depression programmes both include one placebo-controlled and one dose–response study. However, we predict with reasonable confidence that dose–response studies will not solve the identification issue since (1) the studies may not test for functional unblinding as it is not mandated by the FDA^27^ and (2) unblinding will presumably covary with the dose, given confounding the interpretation of results. Pragmatic phase III CE trials would provide complementary evidence to explanatory trial results. If a CE trial were to show superiority to treatment-as-usual, then this would be strong evidence in favour of PAT. However, even demonstration of non-inferiority or equivalence provides some useful evidence, as at the level of an individual patient, approval of PAT would at least expand patient choice. Importantly, pharmacoeconomic evaluation of treatments provides more realistic estimates when based on effectiveness data and reduces the need for complex and potentially unrealistic economic modelling.^87^ For example, recent economic modelling estimates of MDMA-AT for PTSD^37,88^ are based on efficacy and not real-world effectiveness data, leading to great uncertainty in the validity of the estimates. Indeed, it has been proposed that as interventions get more complex, it is more likely that efficacy estimates will tend to be more favourable to the intervention than effectiveness estimates.^89^

More generally, it is important to emphasise that, although strengths and weaknesses of explanatory and pragmatic trials have been known for a long time (e.g. Feinstein^90^), industry regulators have become reliant almost exclusively on data from RCTs, largely dismissing other data sources and experimental designs. For pragmatic trial data to be really useful, policymakers and regulators should engage with recent advancements in the philosophy of evidence in medicine, including but not limited to proposals such as the updating of Evidence-Based Medicine (EBM) to EBM+,^91^ which arguably will be necessary to adequately assess evidence from a diversity of study designs with psychedelic compounds.^14,15^

## Positionality of psychedelic researchers

The choice of methodologies used and questions asked in biomedical research tend to be constrained by the requirements and norms of regulatory and ethical review bodies, investigator training, funding bodies and commercial incentive structures. We end with a structural analysis that suggests academic/community-based researchers are well placed to lead the way in conducting pragmatic trials towards PAT development.

Psychedelic research in this millennium initially re-emerged through several small investigator-initiated trials funded primarily through philanthropic sources. More recently – and particularly since the explosion in hype around psychedelic therapies – this has become a more diverse mixture ranging from industry-led trials hoping to register psychedelics as medicines to a sprawling portfolio of smaller investigator-initiated trials funded by both philanthropic and public sources. Given the requirements for large sample sizes to power explanatory trials outlined above, we consider it unlikely that industry actors will spend significant amounts of capital to also conduct the type of research we suggest here, as they seek to conduct trials that meet extant regulatory requirements and provide returns on investment for stakeholders. Working towards regulatory approval also suggests research strategies that avoid introducing a host of new variables in the research process, which we consider part of the epistemically generative power of pragmatic trials and complex intervention development approaches. One might view a future where the type of research suggested here could reshape regulatory requirements for medicine approval, but getting to this point itself would first require expansion in the use and normalisation of pragmatic drug trials.

By contrast with industry actors, we consider academic/community-based researchers to be particularly well placed to conduct pragmatic over explanatory trials. As noted, there are no IP obstacles to them independently accessing off-patent psychedelics. In addition, academics often bring existing relationships with local community groups and actors together with diverse disciplinary and methodological approaches to the study and improvement of community health. Sometimes these even include research and evaluation approaches specifically tailored to, and co-produced with, local community groups. University-based researchers also bring intellectual curiosity beyond what can be immediately instrumentalised, including reflecting upon their research findings and complicating their theoretical frameworks. There have been many attempts to articulate the potential of the changing role of universities, including through images of the ‘engaged university’^92^ the ‘transformative university’^93^ and the ‘ecological university’.^94^ All offer versions of community–university partnership, with universities serving broader communities. Universities are being imagined as sites for the assembling of new ‘publics’ (e.g. Facer^95^), each made up of diverse stakeholders bound together by some key matter of concern. These visions of the university align with the mission of conducting pragmatic trials embedded within existing community contexts and responding to a particular problem, whether that is problematic drug use, or high rates of depression or PTSD, in the ways that these manifest locally.

We do not mean to paint too rosy a picture of university research. Historically, their relationships with the communities they work amidst have been fraught, and today they are shaped by neoliberal pressures in ways that can compromise meaningful community engagement efforts.^95^ Psychedelic research conducted by academics must also compete for funding – often with other medical research. Medical funding bodies and reviewers might not be familiar with the pragmatic psychedelic trial designs. In today’s funding landscape, these kinds of trials may be more attractive to public health- and community-development-oriented funding bodies seeking immediate community-based health improvements. A final hope of this article is to provide a philosophical position and reference work for those pursuing such funding. Indeed, the subset of larger CER trials that we envisage would require the collaboration of large networks of academics/clinicians working under the same protocol. Although, we are not aware of such efforts yet occurring in psychedelic medicine, such large investigator-led multinational and institutional trials are common in other areas of medicine (e.g. surgery/anaesthesiology).

## Concluding remarks – avoiding history repeating

PAT was pioneered in the 1950s by researchers Humphrey Osmond, Abram Hoffer and Alfred Hubbard as LSD psychotherapy based on the principle that the LSD experience promoted changes in attitude, perspective and behaviour that could be harnessed to facilitate positive treatment outcomes.^96^ In particular, these original developers considered that LSD psychotherapy was not a drug treatment per se but a form of psychotherapy. However, since LSD psychotherapy involved drug administration it came under the purview of the FDA and required evidence of efficacy gathered through controlled (double-blind) clinical trials to be approved as a treatment.^57,97^ While the DB-RCT is well suited for testing simple pharmaceutical products, the application of the DB-RCT method to PAT leads to difficulties as many of the underlying assumptions required for causal inference can be violated.^10,57^ Oram^57^ argues that these conceptual and technical difficulties were a major cause of the decline in PAT research in the 1960’s/1970’s. Pioneering LSD researcher of the time, Sidney Cohen, reflected in his book^98^ ‘*The difficulties of doing a clear-cut study would be far from solved even with these precautions. A control group of patients matched as well as possible with the LSD patients must be given the identical treatment except that LSD is not used. . .. It is quite impossible to keep the therapist in the dark about who is getting the LSD because of its pronounced action. Will he invest as much energy and dedication to his non-LSD patients? The patients themselves will quickly know whether they have received LSD or not. . .. These difficulties and others are the reasons why a decisive test of the efficacy of LSD has not yet been performed. The problems are great but surmountable* (p.199)’. In the 2020s, PAT now finds itself facing similar conceptual difficulties as it did in the 1960s with the limitations of the DB-RCT approach. However, in 70 years since PAT was developed, other methodological approaches to evidence gathering that we have outlined here have been developed. These include complex intervention development, pragmatic trials, comparative effectiveness research, biomarkers, sound clinical pharmacological principles and pre-clinical models (which add mechanistic evidence and reasoning).^15^ By broadening the expanse of evidence gathering for PAT to include these approaches, and in inviting academic researchers to take the lead, we are hopeful that history need not repeat itself.

## Declarations

**Ethics approval and consent to participate:** Not applicable.

**Consent for publication:** Not applicable.

**Availability of data and materials:** Not applicable.

## References

[1] Xylo Bio, https://www.linkedin.com/pulse/update-psychedelic-clinical-trials-psylo-wpinc#:~:text=As%20of%20May%202024%2C%20278,terminated%27%20and%20%27withdrawn%27 (2024, accessed 25 September 2025).

[2] Norring SA Spigarelli MG . The promise of therapeutic psilocybin: an evaluation of the 134 clinical trials, 54 potential indications, and 0 marketing approvals on ClinicalTrials.gov. Drug Des Devel Ther 2024; 18: 1143–1151. 10.2147/DDDT.S443177 PMC11016263 38618282

[3] Skivington K Matthews L Simpson SA , et al. A new framework for developing and evaluating complex interventions: update of Medical Research Council guidance. BMJ 2021; 374: n2061. 10.1136/bmj.n2061 PMC8482308 34593508

[4] Loudon K Treweek S Sullivan F , et al. The PRECIS-2 tool: designing trials that are fit for purpose. BMJ 2015; 350: h2147. 10.1136/bmj.h2147 25956159

[5] National Cancer Institute, https://www.cancer.gov/publications/dictionaries/cancer-terms/def/intervention# (2024, accessed 25 September 2025).

[6] Petticrew M. When are complex interventions ‘complex’? when are simple interventions ‘simple’? Eur J Public Health 2011; 21: 397–398. 21771736 10.1093/eurpub/ckr084

[7] Noorani T Liebert RJ. Real-world evidence of the collective effects of psychedelic therapy: evaluating from the grassroots. J Psychedelic Stud 2024; 8: 260–268.

[8] Skivington K Matthews L Simpson SA , et al. Framework for the development and evaluation of complex interventions: gap analysis, workshop and consultation-informed update. Health Technol Assess 2021; 25: 1–132. 10.3310/hta25570 PMC7614019 34590577

[9] Noorani T Bedi G Muthukumaraswamy S. Dark loops: contagion effects, consistency and chemosocial matrices in psychedelic-assisted therapy trials. Psychol Med 2023; 53(13): 1–10. 10.1017/S0033291723001289 PMC10520581 37466178

[10] Muthukumaraswamy SD. Overcoming blinding confounds in psychedelic randomized controlled trials using biomarker driven causal mediation analysis. Expert Rev Clin Pharmacol 2023; 16(12): 1–11. 37947758 10.1080/17512433.2023.2279736

[11] Deaton A Cartwright N. Understanding and misunderstanding randomized controlled trials. Soc Sci Med 2018; 210: 2–21. 29331519 10.1016/j.socscimed.2017.12.005 PMC6019115

[12] Muthukumaraswamy S Forsyth A Lumley T. Blinding and expectancy confounds in psychedelic randomised controlled trials. Expert Rev Clin Pharmacol 2021; 14: 1133–1152. 34038314 10.1080/17512433.2021.1933434

[13] Kaptchuk TJ. The double-blind, randomized, placebo-controlled trial: gold standard or golden calf? J Clin Epidemiol 2001; 54: 541–549. 11377113 10.1016/s0895-4356(00)00347-4

[14] Schenberg EE. Comment on: history repeating: guidelines to address common problems in psychedelic science. Ther Adv Psychopharmacol 2024; 14: 204512533241243242. 10.1177/20451253241263299 PMC11334119 39166138

[15] Schenberg EE. From efficacy to effectiveness: evaluating psychedelic randomized controlled trials for trustworthy evidence-based policy and practice. Pharmacol Res Perspect 2025; 13: e70097. 10.1002/prp2.70097 PMC11997373 40230191

[16] Muthukumaraswamy S Forsyth A Sumner RL. The challenges ahead for psychedelic ‘medicine’. Aust N Z J Psychiatry 2022; 56(11): 1378–1383. 35243919 10.1177/00048674221081763

[17] Huneke NTM Fusetto Veronesi G Garner M , et al. Expectancy effects, failure of blinding integrity, and placebo response in trials of treatments for psychiatric disorders: a narrative review. JAMA Psychiatry 2025; 82: 531–538. 40072447 10.1001/jamapsychiatry.2025.0085

[18] Mitchell JM Bogenschutz M Lilienstein A , et al. MDMA-assisted therapy for severe PTSD: a randomized, double-blind, placebo-controlled phase 3 study. Nat Med 2021; 27: 1025–1033. 33972795 10.1038/s41591-021-01336-3 PMC8205851

[19] Mitchell JM Ot’alora G M van der Kolk B , et al. MDMA-assisted therapy for moderate to severe PTSD: a randomized, placebo-controlled phase 3 trial. Nat Med 2023; 29: 2473–2480. 37709999 10.1038/s41591-023-02565-4 PMC10579091

[20] Hennink M Kaiser BN. Sample sizes for saturation in qualitative research: a systematic review of empirical tests. Soc Sci Med 2022; 292: 114523. 34785096 10.1016/j.socscimed.2021.114523

[21] Dworkin RH McDermott MP Nayak SM , et al. Psychedelics and psychotherapy: is the whole greater than the sum of its parts? Clin Pharmacol Ther 2023; 114: 1166–1169. 37795632 10.1002/cpt.3050

[22] Langlitz N Gearin AK. Psychedelic therapy as form of life. Neuroethics 2024; 17: 14.

[23] Schwartz D Lellouch J. Explanatory and pragmatic attitudes in therapeutical trials. J Chronic Dis 1967; 20: 637–648. 4860352 10.1016/0021-9681(67)90041-0

[24] Hotopf M. The pragmatic randomised controlled trial. Adv Psychiatr Treat 2002; 8: 326–333.

[25] Gartlehner G Hansen RA Nissman D , et al. Criteria for distinguishing effectiveness from efficacy trials in systematic reviews. April : Report No 06–0046. 2010. 20734508

[26] Food and Drug Administration. Demonstrating substantial evidence of effectiveness for human drug and biological products. https://www.fda.gov/regulatory-information/search-fda-guidance-documents/demonstrating-substantial-evidence-effectiveness-human-drug-and-biological-products. (2019, accessed 25 September 2025).

[27] Food and Drug Administration. Psychedelic drugs: considerations for clinical investigations. https://www.fda.gov/regulatory-information/search-fda-guidance-documents/psychedelic-drugs-considerations-clinical-investigations (2023, accessed25 September 2025).

[28] Food and Drug Administration. https://www.fda.gov/advisory-committees/advisory-committee-calendar/updated-meeting-time-and-public-participation-information-june-4-2024-meeting-psychopharmacologic (2024, accessed 25 September 2025).

[29] Ford I Norrie J. Pragmatic trials. N Engl J Med 2016; 375: 454–463. 27518663 10.1056/NEJMra1510059

[30] Thorpe KE Zwarenstein M Oxman AD , et al. A pragmatic-explanatory continuum indicator summary (PRECIS): a tool to help trial designers. J Clin Epidemiol 2009; 62: 464–475. 19348971 10.1016/j.jclinepi.2008.12.011

[31] Carhart-Harris RL Wagner AC Agrawal M , et al. Can pragmatic research, real-world data and digital technologies aid the development of psychedelic medicine? J Psychopharmacol 2022; 36: 6–11. 33888025 10.1177/02698811211008567 PMC8801625

[32] Daldegan-Bueno D Donegan CJ Forsyth A et al. LSDDEP2: study protocol for a randomised double-dummy triple-blind active placebo-controlled parallel groups trial of LSD microdosing in patients with major depressive disorder. Trials 2024; 25(1): 560. 39182140 10.1186/s13063-024-08384-3 PMC11344334

[33] Halman A Kong G Sarris J , et al. Drug-drug interactions involving classic psychedelics: a systematic review. J Psychopharmacol 2024; 38: 3–18. 37982394 10.1177/02698811231211219 PMC10851641

[34] Sarparast A Thomas K Malcolm B , et al. Drug-drug interactions between psychiatric medications and MDMA or psilocybin: a systematic review. Psychopharmacol (Berl) 2022; 239: 1945–1976. 10.1007/s00213-022-06083-y PMC9177763 35253070

[35] Wolfgang AS Fonzo GA Gray JC , et al. MDMA and MDMA-assisted therapy. Am J Psychiatry 2025; 182: 79–103. 39741438 10.1176/appi.ajp.20230681

[36] Honk L Stenfors CUD Goldberg SB , et al. Longitudinal associations between psychedelic use and psychotic symptoms in the United States and the United Kingdom. J Affect Disord 2024; 351: 194–201. 38280572 10.1016/j.jad.2024.01.197 PMC10922895

[37] Marseille E Mitchell JM Kahn JG. Updated cost-effectiveness of MDMA-assisted therapy for the treatment of posttraumatic stress disorder in the United States: findings from a phase 3 trial. PLoS One 2022; 17: e0263252. 10.1371/journal.pone.0263252 PMC8880875 35213554

[38] Marseille E Stauffer CS Agrawal M , et al. Group psychedelic therapy: empirical estimates of cost-savings and improved access. Front Psychiatry 2023; 14: 1293243. 38125286 10.3389/fpsyt.2023.1293243 PMC10731307

[39] Welsing PM Oude Rengerink K Collier S , et al. Series: pragmatic trials and real world evidence: paper 6. outcome measures in the real world. J Clin Epidemiol 2017; 90: 99–107. 28502810 10.1016/j.jclinepi.2016.12.022

[40] Carhart-Harris R Giribaldi B Watts R , et al. Trial of psilocybin versus escitalopram for depression. N Engl J Med 2021; 384: 1402–1411. 33852780 10.1056/NEJMoa2032994

[41] Goodwin GM Aaronson ST Alvarez O , et al. Single-dose psilocybin for a treatment-resistant episode of major depression. N Engl J Med 2022; 387: 1637–1648. 36322843 10.1056/NEJMoa2206443

[42] Raison CL Sanacora G Woolley J , et al. Single-dose psilocybin treatment for major depressive disorder: a randomized clinical trial. JAMA 2023; 330: 843–853. 37651119 10.1001/jama.2023.14530 PMC10472268

[43] Holze F Gasser P Müller F , et al. Lysergic acid diethylamide-assisted therapy in patients with anxiety with and without a life-threatening illness: a randomized, double-blind, placebo-controlled phase II study. Biol Psychiatry 2023; 93: 215–223. 36266118 10.1016/j.biopsych.2022.08.025

[44] Purgato M Barbui C Stroup S , et al. Pragmatic design in randomized controlled trials. Psychol Med 2015; 45: 225–230. 25065958 10.1017/S0033291714001275

[45] Patsopoulos NA. A pragmatic view on pragmatic trials. Dialogues Clin Neurosci 2011; 13: 217–224. 21842619 10.31887/DCNS.2011.13.2/npatsopoulos PMC3181997

[46] Schenberg EE King F IV da Fonseca JE , et al. Is poorly assisted psilocybin treatment an increasing risk? Am J Psychiatry 2024; 181: 75–76. 10.1176/appi.ajp.20230664 38161296

[47] Earleywine M Leo JD Bhayana D , et al.Psilocybin without psychotherapy: a cart without a horse? Am J Psychiatry 2024; 181: 78–79. 38161299 10.1176/appi.ajp.20230572

[48] Alpert MD O’Donnell KC Paleos CA , et al. Psychotherapy in psychedelic treatment: safe, evidence-based, and necessary. Am J Psychiatry 2024; 181: 76–77. 38161307 10.1176/appi.ajp.20230665

[49] Goodwin GM Malievskaia E Fonzo GA , et al. Must psilocybin always ‘Assist Psychotherapy’? Am J Psychiatry 2024; 181: 20–25. 37434509 10.1176/appi.ajp.20221043

[50] Gründer G Brand M Mertens LJ , et al. Treatment with psychedelics is psychotherapy: beyond reductionism. Lancet Psychiatry 2024; 11: 231–236. 38101439 10.1016/S2215-0366(23)00363-2

[51] Tai SJ Nielson EM Lennard-Jones M , et al. Development and evaluation of a therapist training program for psilocybin therapy for treatment-resistant depression in clinical research. Front Psychiatry 2021; 12: 586682. 10.3389/fpsyt.2021.586682 PMC7908919 33643087

[52] Aday JS Barnett BS Grossman D , et al. Psychedelic commercialization: a wide-spanning overview of the emerging psychedelic industry. Psychedelic Med 2023; 1: 150–165. 10.1089/psymed.2023.0013 PMC11661494 40046566

[53] Langlitz N. Psychedelic innovations and the crisis of psychopharmacology. BioSocieties 2024; 19: 37–58.

[54] Aday JS Heifets BD Pratscher SD , et al. Great expectations: recommendations for improving the methodological rigor of psychedelic clinical trials. Psychopharmacol (Berl) 2022; 239: 1989–2010. 10.1007/s00213-022-06123-7 PMC10184717 35359159

[55] Goodwin GM. Psilocybin: psychotherapy or drug? J Psychopharmacol 2016; 30: 1201–1202. 27909167 10.1177/0269881116675757

[56] Schenberg EE. Who is blind in psychedelic research? letter to the editor regarding: blinding and expectancy confounds in psychedelic randomized controlled trials. Expert Rev Clin Pharmacol 2021; 14: 1317–1319. 34227438 10.1080/17512433.2021.1951473

[57] Oram M. The trials of psychedelic therapy: LSD psychotherapy in America. Baltimore: John Hopkins University Press, 2018.

[58] Grünbaum A. The placebo concept in medicine and psychiatry. Psychol Med 1986; 16: 19–38. 3515378 10.1017/s0033291700002506

[59] Howick J. The relativity of ‘placebos’: defending a modified version of Grünbaum’s definition. Synthese 2017; 194: 1363–1396.

[60] Gaab J Locher C Blease C. Chapter thirteen – placebo and psychotherapy: differences, similarities, and implications. In: Colloca L (ed.) International review of neurobiology. Cambridge, MA: Academic Press, 2018, pp.241–255. 10.1016/bs.irn.2018.01.013 29681328

[61] Blease CR. Psychotherapy and placebos: manifesto for conceptual clarity. Front Psychiatry 2018; 9: 379. 30177892 10.3389/fpsyt.2018.00379 PMC6109685

[62] Wampold BE. How important are the common factors in psychotherapy? An update. World Psychiatry 2015; 14: 270-277. DOI: 10.1002/wps.20238. 26407772 PMC4592639

[63] Grawe K. Research-informed psychotherapy. Psychother Res 1997; 7: 1–19.

[64] Grawe K. Psychological therapy. Cambridge, MA: Hogrefe Publishing GmbH, 2004.

[65] Gukasyan N Nayak SM. Psychedelics, placebo effects, and set and setting: insights from common factors theory of psychotherapy. Transcult Psychiatry 2022; 59: 652–664. 33499762 10.1177/1363461520983684

[66] Wolff M Evens R Mertens LJ , et al. Measuring psychotherapeutic processes in the context of psychedelic experiences: validation of the General Change Mechanisms Questionnaire (GCMQ). J Psychopharmacol 2024; 38: 432–457. 38742761 10.1177/02698811241249698 PMC11102652

[67] Gaab J Blease C Locher C , et al. Go open: a plea for transparency in psychotherapy. Psychol Conscious: Theory, Research, and Practice 2016; 3: 175–198.

[68] Gold SM Enck P Hasselmann H , et al. Control conditions for randomised trials of behavioural interventions in psychiatry: a decision framework. Lancet Psychiatry 2017; 4: 725–732. 28396067 10.1016/S2215-0366(17)30153-0

[69] Haaga DAF Stiles WB . Randomized clinical trials in psychotherapy research: methodology, design, and evaluation. In: Snyder CR Ingram RE (eds) Handbook of psychological change: psychotherapy processes & practices for the 21st century. Hoboken, NJ: John Wiley & Sons, Inc., 2000, pp.14–39.

[70] Mohr DC Spring B Freedland KE , et al. The selection and design of control conditions for randomized controlled trials of psychological interventions. Psychother Psychosom 2009; 78: 275–284. 19602916 10.1159/000228248

[71] Weimer K Enck P. Traditional and innovative experimental and clinical trial designs and their advantages and pitfalls. In: Benedetti F Enck P Frisaldi E , et al. (eds) Placebo. Berlin, Heidelberg: Springer, 2014, pp.237–272. 10.1007/978-3-662-44519-8_14 25304536

[72] Mohr DC Ho J Hart TL , et al. Control condition design and implementation features in controlled trials: a meta-analysis of trials evaluating psychotherapy for depression. Transl Behav Med 2014; 4: 407–423. 25584090 10.1007/s13142-014-0262-3 PMC4286544

[73] Wampold BE Mondin GW Moody M , et al. A meta-analysis of outcome studies comparing bona fide psychotherapies: empiricially, ‘all must have prizes.’ Psychol Bull 1997; 122: 203–215.

[74] Kirsch I Wampold BE Kelley JM. Controlling for the placebo effect in psychotherapy: noble quest or tilting at windmills? Psychol Consciousn Theory Res Pract 2016; 3(2): 121–131.

[75] Cristea IA Cuijpers P Halvorsen JØ . The psychotherapy in MDMA-assisted psychotherapy. JAMA Psychiatry 2024; 81: 1053–1054. 39356536 10.1001/jamapsychiatry.2024.2887

[76] Lemarchand C Chopin R Paul M , et al. Fragile promise of psychedelics in psychiatry. BMJ 2024; 387: e080391. 10.1136/bmj-2024-080391 39562018

[77] Cristea IA Halvorsen JØ Cosgrove L , et al. New treatments for mental disorders should be routinely compared to psychotherapy in trials conducted for regulatory purposes. Lancet Psychiatry 2022; 9: 934–936. 36403594 10.1016/S2215-0366(22)00340-6

[78] Klein DF. Preventing hung juries about therapy studies. J Consult Clin Psychol 1996; 64: 81–87. 8907087 10.1037//0022-006x.64.1.81

[79] Juul S Gluud C Simonsen S , et al. Blinding in randomised clinical trials of psychological interventions: a retrospective study of published trial reports. BMJ Evid Based Med 2021; 26: 109. 10.1136/bmjebm-2020-111407 32998993

[80] Jonsson U Alaie I Parling T , et al. Reporting of harms in randomized controlled trials of psychological interventions for mental and behavioral disorders: a review of current practice. Contemp Clin Trials 2014; 38: 1–8. 24607768 10.1016/j.cct.2014.02.005

[81] Linden M Schermuly-Haupt ML. Definition, assessment and rate of psychotherapy side effects. World Psychiatry 2014; 13: 306–309. 25273304 10.1002/wps.20153 PMC4219072

[82] Fonagy P Luyten P. Fidelity vs. Flexibility in the implementation of psychotherapies: time to move on. World Psychiatry 2019; 18: 270–271. 31496081 10.1002/wps.20657 PMC6732688

[83] Peyser D Sysko R Webb L , et al. Treatment fidelity in eating disorders and psychological research: current status and future directions. Int J Eat Disord 2021; 54: 2121–2131. 34622960 10.1002/eat.23624 PMC8719268

[84] Sox HC Goodman SN. The methods of comparative effectiveness research. Annu Rev Public Health 2012; 33: 425–445. 22224891 10.1146/annurev-publhealth-031811-124610

[85] Compass Pathways, https://s204.q4cdn.com/927483861/files/doc_presentation/2024/11/COMPASS-Pathways-investor-presentation_Nov-2024-_post-Q3.pdf (2024, accessed 25 September 2025).

[86] Cybin Inc, https://ir.cybin.com/investors/news/news-details/2024/Cybin-Initiates-PARADIGM-A-Multinational-Pivotal-Phase-3-Program-Evaluating-CYB003-for-the-Adjunctive-Treatment-of-Major-Depressive-Disorder-and-Reports-Second-Quarter-Financial-Results/ (2024, accessed 25 September 2025).

[87] Bombardier C Maetzel A. Pharmacoeconomic evaluation of new treatments: efficacy versus effectiveness studies? Ann Rheum Dis 1999; 58(Suppl 1): 182–185. 10577979 10.1136/ard.58.2008.i82 PMC1766572

[88] Mustafa R McQueen B Nikitin D , et al. MDMA-assisted psychotherapy for post-traumatic stress disorder: effectiveness and value. Final Evidence Report. Institute for Clinical and Economic Review, June 2024.

[89] Nallamothu BK Hayward RA Bates ER. Beyond the randomized clinical trial. Circulation 2008; 118: 1294–1303. 18794402 10.1161/CIRCULATIONAHA.107.703579

[90] Feinstein AR . An additional basic science for clinical medicine: II. The limitations of randomized trials. Ann Intern Med 1983; 99: 544–550. 6625387 10.7326/0003-4819-99-4-544

[91] Aronson JK La Caze A Kelly MP , et al. The use of mechanistic evidence in drug approval. J Eval Clin Pract 2018; 24: 1166–1176. 29888417 10.1111/jep.12960 PMC6175306

[92] Watson D Hollister R Stroud SE , et al. The engaged university: international perspectives on civic engagement. New York: Routledge, 2011.

[93] Guzmán-Valenzuela C. Unfolding the meaning of public (s) in universities: toward the transformative university. Higher Education 2016; 71: 667–679.

[94] Barnett R. The ecological university: A feasible Utopia. Abingdon: Routledge, 2017.

[95] Facer K. 2. Convening publics? co-produced research in the entrepreneurial university. Philosophy and Theory in Higher Education 2020; 2: 19–43.

[96] Oram M. Efficacy and enlightenment: LSD psychotherapy and the drug amendments of 1962. Journal of the History of Medicine and Allied Sciences 2012; 69: 221–250. 22898355 10.1093/jhmas/jrs050

[97] Bonson KR. Regulation of human research with LSD in the United States (1949-1987). Psychopharmacology (Berl) 2018; 235: 591–604. 29147729 10.1007/s00213-017-4777-4

[98] Cohen S . The beyond within the LSD story. New York: Atheneum, 1964.

