# Corrigendum to “Psychedelic Assisted Therapy as a Complex Intervention: Implications for clinical trial design”

**DOI:** 10.1177/20451253251395232

**Published:** 2025-11-09

Muthukumaraswamy SD, Baggott MJ, Schenberg EE et al. Psychedelic Assisted Therapy as a Complex Intervention: Implications for clinical trial design. *Therapeutic Advances in Psychopharmacology* 2025; 15: 1–19. doi: 10.1177/20451253251381074

In this article, the first author’s name was incorrectly listed as “S. D. Muthumaraswamy”. The online version has been updated to reflect the correct name, “S. D. Muthukumaraswamy”.

